# Supplementary material for: Single-cell imaging of protein dynamics of paralogs reveals mechanisms of gene retention
Source: bioRxiv. 2023 Nov 23:2023.11.23.568466. Preprint. [Version 1] doi: 10.1101/2023.11.23.568466 (PMC10690282; doi:10.1101/2023.11.23.568466)
Supplement: Supplement 1 [file NIHPP2023.11.23.568466v1-supplement-1.pdf]

bioRxiv preprint doi: https://doi.org/10.1101/2023.11.23.568466; this version posted November 23, 2023. The copyright holder for this preprint (which was not certified by peer review) is the author/funder, who has granted bioRxiv a license to display the preprint in perpetuity. It is made available under aCC-BY 4.0 International license.

Supplementary figures

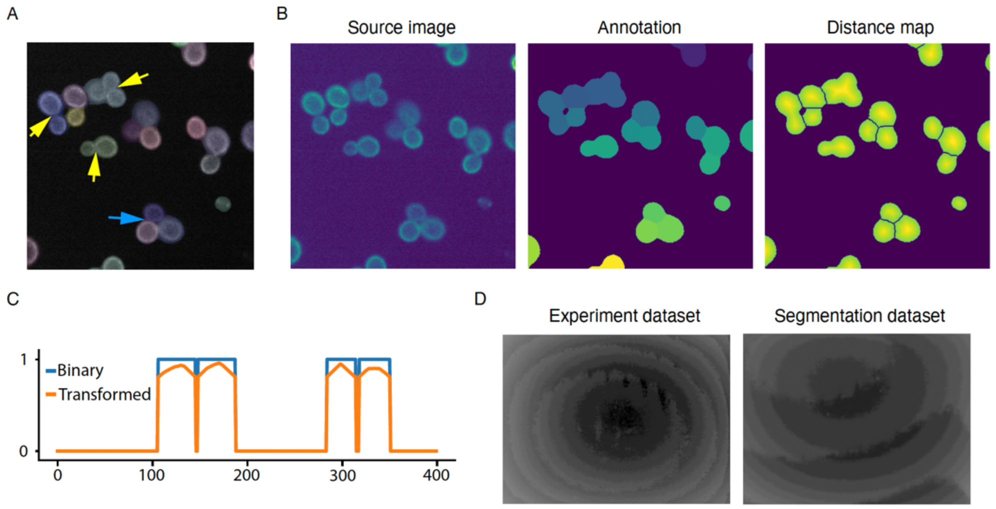

Figure S1. Optimization of the image segmentation.

A. A representative cropped microscopy image, with object annotation overlayed. The yeast cells in the ground truth are shown using yellow arrows. An example of fully separated cells is shown using the blue arrow.

B. Illustration of the proposed annotation processing into distance maps. Left: cropped microscopy image; center: ground truth object annotation; right: output distance map.

C. Vertical cross-sections of the binary segmentation and the distance map.

D. Log-transformed read-out noise highlights the uneven illumination pattern in both datasets.

bioRxiv preprint doi: https://doi.org/10.1101/2023.11.23.568466; this version posted November 23, 2023. The copyright holder for this preprint (which was not certified by peer review) is the author/funder, who has granted bioRxiv a license to display the preprint in perpetuity. It is made available under aCC-BY 4.0 International license.

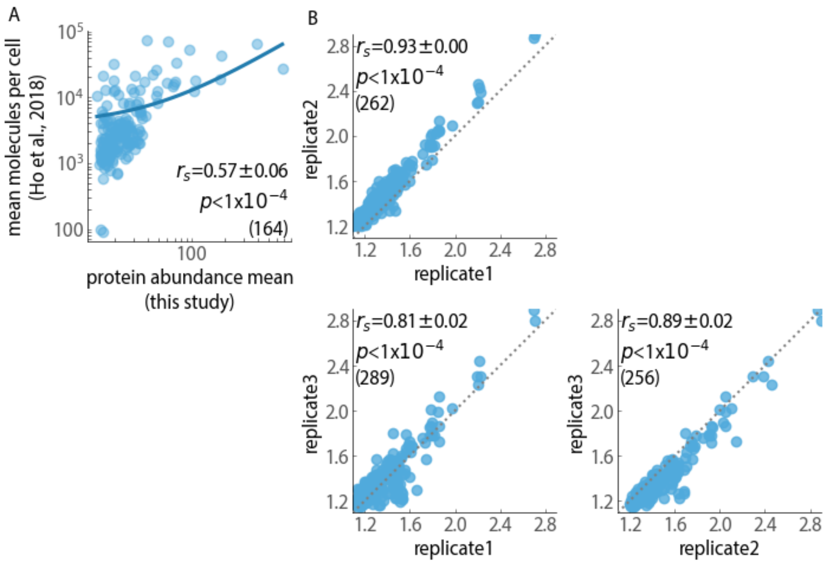

Figure S2. Protein abundance conformity with the known standard and reproducibility across replicate measurements.

A. Correlation of the abundance values in the wild-type background with the reference values (Ho, Baryshnikova, and Brown 2018). The line indicates the fitted regression model of order 1.

B. Correlations of the protein abundance values (log_{10}-scaled) in the wild-type and deletion backgrounds across replicates. r_s: Spearman's rank correlation coefficient; p: p-value associated with the correlation. The robustness of the correlations was tested by five rounds of resampling.

bioRxiv preprint doi: https://doi.org/10.1101/2023.11.23.568466; this version posted November 23, 2023. The copyright holder for this preprint (which was not certified by peer review) is the author/funder, who has granted bioRxiv a license to display the preprint in perpetuity. It is made available under aCC-BY 4.0 International license.

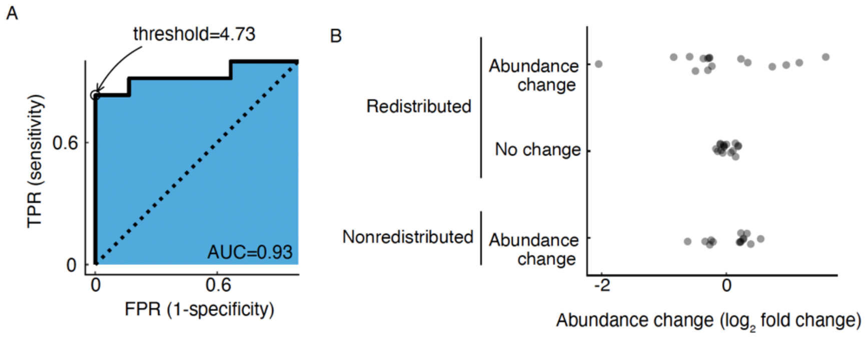

Figure S3. The classification of redistributed paralogs and comparison with the relative abundance change.

A. Receiver Operating Characteristic (ROC) curve for the classification of the redistributed paralogs.

B. Relative abundance change scores of paralogs stratified by the significance of redistribution and abundance change.

bioRxiv preprint doi: https://doi.org/10.1101/2023.11.23.568466; this version posted November 23, 2023. The copyright holder for this preprint (which was not certified by peer review) is the author/funder, who has granted bioRxiv a license to display the preprint in perpetuity. It is made available under aCC-BY 4.0 International license.

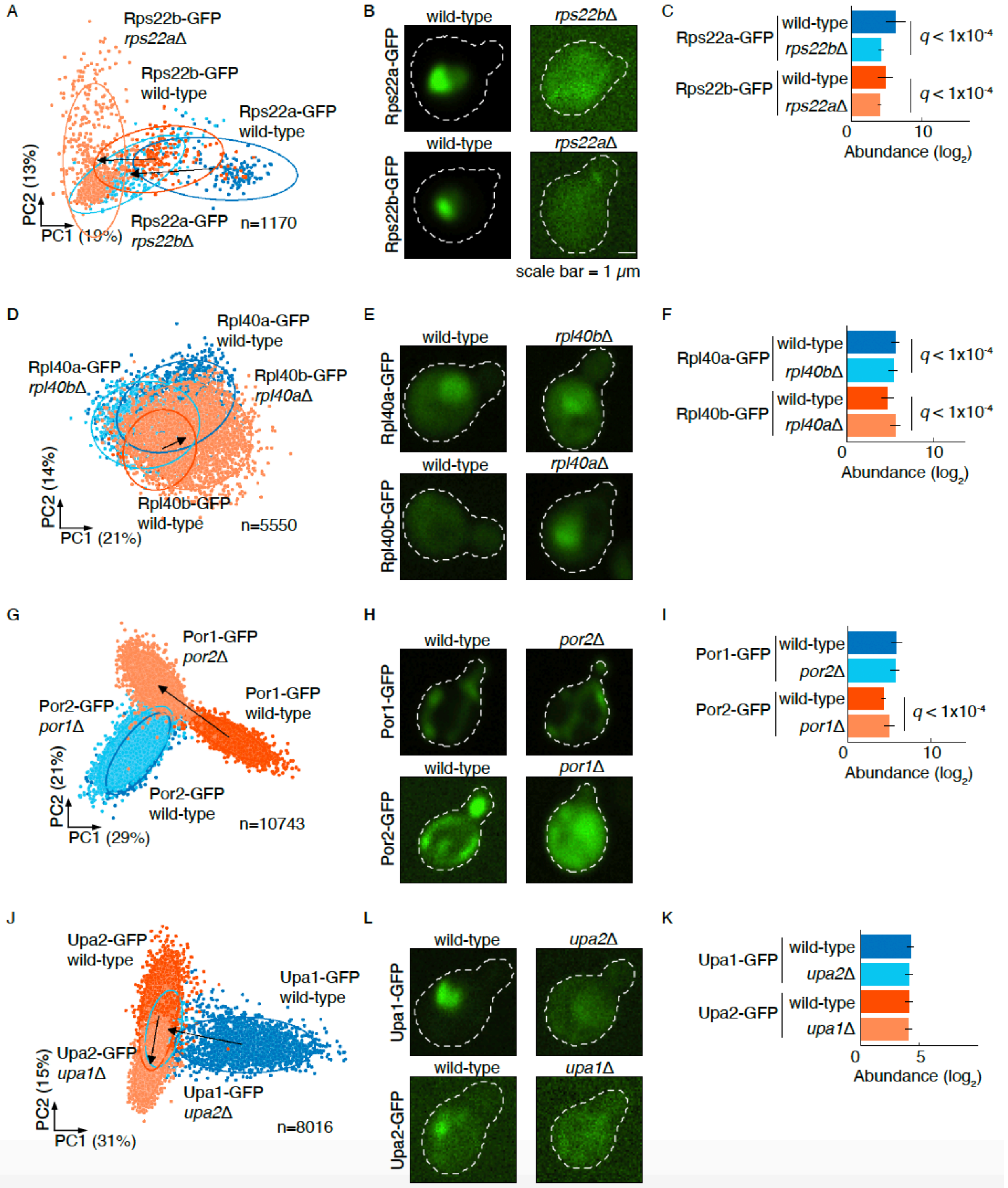

Figure S4. Redistribution, relative abundance and relocalization of paralog pairs.

A. Redistribution of the Rps22a-Rps22b (in **A-C**), Rpl40a-Rpl40b (**D-F**), Por1-Por2 (**G-I**) and Upa1-Upa2 (**J-L**) pairs. Principal component analysis (PCA) of z-score normalized features is shown for each construct. For the redistributed paralog, the arrow connects the centroids of the clusters corresponding to the wild-type (dark blue/orange) and deletion (light

bioRxiv preprint doi: https://doi.org/10.1101/2023.11.23.568466; this version posted November 23, 2023. The copyright holder for this preprint (which was not certified by peer review) is the author/funder, who has granted bioRxiv a license to display the preprint in perpetuity. It is made available under aCC-BY 4.0 International license.

blue/orange) backgrounds of the sister paralog. The percentage variances explained by the PCs are indicated in parentheses.

B. Micrographs of representative yeast cells of respective paralog pairs.

C. Relative abundance changes of the respective pairs. The error bars show the 95% confidence intervals of the means. *q*: FDR-corrected *p*-value. Wild-type (dark blue/orange) and deletion (light blue/orange) backgrounds of the paralog.

bioRxiv preprint doi: https://doi.org/10.1101/2023.11.23.568466; this version posted November 23, 2023. The copyright holder for this preprint (which was not certified by peer review) is the author/funder, who has granted bioRxiv a license to display the preprint in perpetuity. It is made available under aCC-BY 4.0 International license.

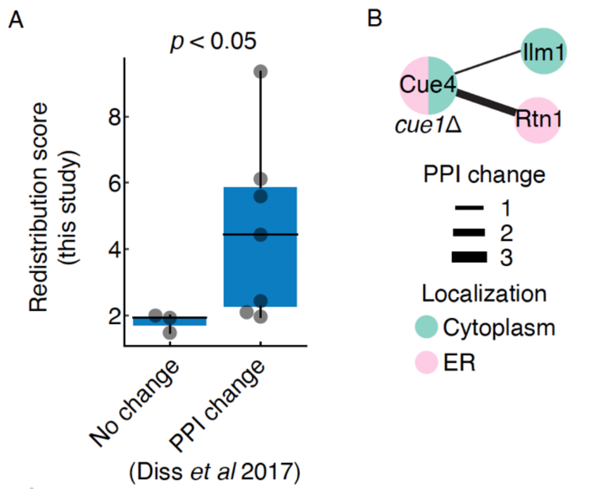

Figure S5. Redistributed paralogs show change in protein-protein interactions (PPIs).

A. The comparison of the redistribution scores (this study) for proteins that show no change in PPIs with those that show at least one PPI change when their paralog is deleted. Statistical significance was determined using a two-sided Mann-Whitney U-test.

B. Cue1-Cue4 represent an example of paralogous proteins in which only Cue4 shows redistribution (this study) and a corresponding PPI change upon *CUE1* deletion. The colors of the nodes indicate the subcellular compartments. Edges connect interacting proteins; edge thickness shows PPI change.

PPI change was obtained from a previous study (Diss et al. 2017). In the boxplots, the central line indicates the median, the extent of the box is from the first quartile (Q1) to the third quartile (Q3), and the whiskers extend to Q1-1.5*IQR and Q3+1.5*IQR.

bioRxiv preprint doi: https://doi.org/10.1101/2023.11.23.568466; this version posted November 23, 2023. The copyright holder for this preprint (which was not certified by peer review) is the author/funder, who has granted bioRxiv a license to display the preprint in perpetuity. It is made available under aCC-BY 4.0 International license.

Supplementary tables

Table S1: Yeast strains and plasmids used in this study.
Table S2: Manual inspection of paralog pairs and negative controls.
Table S3: Protein abundance
Table S4: The redistribution, relative abundance change and relocalization.
Table S5: Features of the paralogs.

Supplementary data

Data S1: Features of single cells.
Data S2: The abundance per single cell.
